# Immunotherapies targeting GPRC5D in relapsed or refractory multiple myeloma: latest updates from 2022 ASH Annual Meeting

**DOI:** 10.1186/s13045-023-01461-1

**Published:** 2023-06-05

**Authors:** Jieyun Xia, Zhenyu Li, Kailin Xu

**Affiliations:** Department of Hematology, the Affiliated Hospital of Xuzhou Medical University. Jiangsu Key Laboratory of Bone Marrow Stem Cells. Blood Diseases Institute, Xuzhou Medical University, Xuzhou, China

**Keywords:** GPRC5D, CAR-T, CAR-NK, BiTE, MM

## Abstract

B cell maturation antigen (BCMA)-targeted immunotherapy has shown unprecedented results in the treatment of relapsed or refractory (R/R) multiple myeloma (MM). However, disease progression remains an issue attributed to variable BCMA expression, BCMA downregulation, and heterogeneity of tumor antigens in MM. Therefore, additional treatment options with novel therapeutic targets are warranted. G protein-coupled receptor, class C group 5 member D (GPRC5D), an orphan receptor expressed on malignant plasma cells with limited expression in normal tissue, has emerged as a promising therapeutic target for R/R MM. GPRC5D-targeted chimeric antigen receptor (CAR)-T and CAR-NK cell therapy, as well as bispecific T cell engagers, offer remarkable anti-tumor activities. We summarized some latest reports on GPRC5D-targeted treatments for R/R MM from the 2022 ASH Annual Meeting (ASH 2022).

## To the editor

 The prognosis of patients with relapsed or refractory (R/R) multiple myeloma (MM) is generally poor, and new therapeutic methods are urgently demanded. G protein-coupled receptor family C group 5 member D (GPRC5D) is primarily expressed on myeloma cells, and normal tissue expression is limited to the hair follicle, making it a promising therapeutic target for patients with MM [1–4]. We summarized some impressive developments in GPRC5D-targeted immunotherapies for R/R MM from the 2022 ASH Annual Meeting (ASH 2022).

## GPRC5D CAR-T cell therapy in R/R MM

In patients with R/R MM, anti-GPRC5D chimeric antigen receptor (CAR)-T cell treatment exhibited encouraging clinical efficacy and a manageable safety profile [2–4]. Dr. Bal reported the results of BMS-986393 (CC-95266) trial[5], a phase 1 first-in-human GPRC5D-targeted CAR-T cell therapy in patients with R/R MM (Table 1). In this heavily pretreated population (median four prior therapies, 90% previous transplant, 41% previous B cell maturation antigen [BCMA] -targeted therapies), the initial (1-month) overall response rate (ORR) was 86% (12/14), including 4/6 patients treated with previous BCMA-targeted therapies. No grade ≥ 3 cytokine release syndrome (CRS), on-target/off-tumor activity, or immune effector cell-associated neurotoxicity syndrome (ICANS) events were presented (Table 2).

**Table 1:** Properties of GPRC5D-targeted agents in MM

Author	Agent	Clinical Trial Identifier	Phase	Target	Mechanism	Cell source	References
Sham Mailankody et al.	MCARH109	NCT04555551	1	GPRC5D	CAR-T	Autologous	[2]
Jieyun Xia et al.	GPRC5D CAR-T	ChiCTR2100048888	2	GPRC5D	CAR-T	Autologous	[3]
Mingming Zhang et al.	OriCAR-017	NCT05016778	1	GPRC5D	CAR-T	Autologous	[4]
Susan Bal et al.	BMS-986393	NCT04674813	1	GPRC5D	CAR-T	Autologous	[5]
Ajai Chari et al.	Tal (JNJ-64407564)	NCT03399799/NCT04634552	1/2	GPRC5D,CD3	BiTE	Off-the-shelf	[6]
Yaël C. Cohen et al.	Tal (JNJ-64407564) -DP or Tal-D versus DPd	NCT05455320	3	GPRC5D,CD3	BiTE	Off-the-shelf	[7]
Carmelo Carlo-Stella et al.	RG6234	NCT04557150	1	GPRC5D,CD3	BiTE	Off-the-shelf	[9]
John Reiser	FT555	–	Preclinical	GPRC5D,CD38	CAR-NK	iPSCs master cell line	[11]

*BCMA* B cell maturation antigen, *BiTE* bispecific T cell engager, *CAR* chimeric antigen receptor, *D* daratumumab, *d* dexamethasone, *GPRC5D* G protein-coupled receptor family C group 5 member D, *iPSC* induced pluripotent stem cells, *P* pomalidomide, *Tal* talquetamab

**Table 2:** Outcomes of GPRC5D-targeted clinical trials in MM

Author	Agent	Clinical Trial Identifier	Patients (n)	Medium number of prior LOT	Prior BCMA directed therapy	ORR	≥ CR	ORR in patients with Prior BCMA directed therapy	Grade ≥ 3 CRS	Grade ≥ 3 ICANS	On-target/off-tumor	Nail disorders	References
Sham Mailankody et al.	MCARH109	NCT04555551	17	6	59%	71%	35%	70%	6%	6%	–	65%	[2]
Jieyun Xia et al.	GPRC5D CAR-T	ChiCTR2100048888	33	4	27%	91%	64%	100%	0%	3%	–	27%	[3]
Mingming Zhang et al.	OriCAR-017	NCT05016778	10	5.5	50%	100%	60%	100%	0%	0%	–	30%	[4]
Susan Bal et al.	BMS-986393	NCT04674813	17	4	41%	86%	–	67%	0%	0%	29%	12%	[5]
Ajai Chari et al.	Tal (JNJ-64407564)	NCT03399799/NCT04634552	0.4 mg/kg weekly:143; 0.8 mg/kg biweekly:145	5	–	0.4 mg/kg weekly:73%; 0.8 mg/kg biweekly:74%	0.4 mg/kg weekly:29%	–	0.4 mg/kg weekly:2%; 0.8 mg/kg biweekly:1%	–	–	0.4 mg/kg weekly:52%; 0.8 mg/kg biweekly:43%	[6]
Carmelo Carlo-Stella et al.	RG6234	NCT04557150	IV: 51; SC: 54	IV: 5; SC: 4	IV: 19.6%; SC: 20.4%	IV: 71.4%; SC: 60.4%	IV: 28.5%; SC: 18.8%	55.6%	IV: 2.0%; SC: 1.9%	1.90%	IV: 72.5%; SC: 81.5% (cutaneous AEs)	IV: 17.6%; SC: 22.2%, (hair and nail changes)	[9]

*AEs* adverse events, *CR* complete response, *CRS* cytokine release syndrome, *ICANS* immune effector cell-associated neurotoxicity syndrome, *IV* intravenous, *LOT* lines of therapy, *ORR* overall response rate, *SC* subcutaneous, *T*al talquetamab

Besides GPRC5D CAR-T cell monotherapy, BCMA and GPRC5D dual-target CAR-T cell therapies (NCT05509530, NCT05325801), concurrent administration of GPRC5D-targeted CAR-T cells and BCMA-targeted CAR-T cells (NCT05431608) in patients with R/R MM are being investigated in clinical settings.

## GPRC5D x CD3 bispecific T cell engagers (BiTEs) in R/R MM

Talquetamab (JNJ-64407564) is a first-in-class, off-the-shelf, bispecific T cell engager antibody that targets both GPRC5D and CD3 (Table 1). In the phase 1/2 MonumenTAL-1 study (NCT03399799/NCT04634552) [6], measurable improvement of cancer was observed in 73% of patients receiving 0.4 mg/kg of talquetamab weekly and 74% of patients receiving 0.8 mg/kg every other week, with 29% achieving a complete response. The most common adverse events (AEs) at 0.4 mg/kg weekly /0.8 mg/kg biweekly dose were CRS (79%/72%; grade 3: 2%/1%); skin-related AEs occurred in 56%/68% and nail disorders in 52%/43% of patients (Table 2). In addition, the MonumenTAL-3 trial (NCT05455320) will compare the efficacy and safety of talquetamab plus daratumumab (with or without pomalidomide) with those of daratumumab plus pomalidomide and dexamethasone in patients with RRMM who received ≥ 1 prior line of therapy [7] (Table 1).

RG6234 is another exciting novel GPRC5DxCD3 BiTE[8–10]. Carlo-Stella et al. presented the initial results of an ongoing phase I study (NCT04557150; Table 1, Table 2) [9]. The median number of previous lines of therapy was five in the intravenous (IV) cohorts and four in the subcutaneous (SC) cohorts. Some patients had received BCMA-targeted therapies previously (IV: 19.6%; SC: 20.4%). Clinical activity was observed in both routes of dose escalation (ORR: IV 71.4%, SC 60.4%), including 55.6% of patients who had received previous BCMA-targeted therapies. The drug was well tolerated; CRS and ICANS > grade 2 were both ≤ 2%, and only two patients (3.9%) in the IV group and two patients (3.7%) in the SC group discontinued treatment due to RG6234-related AEs. Biomarker analysis demonstrated rapid T cell activation and T cell-mediated anti-myeloma activity independent of the route of administration [10].

Several GPRC5D CAR-T cell studies reported higher ORR (86%-100%) compared with GPRC5DxCD3 BiTEs (60.4%-74%); CR rates of GPRC5D CAR-T were also higher [2–6, 9] (Table 2). However, the frequency and severity of CRS and ICANS were similar in both the treatments.

## GPRC5D CAR-NK Cell therapy in MM

FT555 is a multiplexed-engineered GRPC5D CAR-NK cell derived from an induced pluripotent stem cells (iPSC) master cell line[11] (Table 1). Compared to isogenic GPRC5D knockout targets, FT555 exhibited persistent specific anti-tumor activity against GPRC5D-positive myeloma cells in the preclinical study. In the disseminated in vivo xenograft model of MM, a single dose of FT555 showed robust killing kinetics and tumor clearance, controlled disease progression for up to 42 days and improved survival to 80 days compared to the untreated control arm of 37 days. The durability of FT555 was further strengthened by the addition of daratumumab, an anti-CD38 monoclonal antibody. Also, tumor growth inhibition was enhanced and survival was significantly prolonged.

In conclusion, the ASH 2022 Annual Meeting exhibited notable advances in the field of GPRC5D-targeted therapies in MM, as summarized in Tables 1 and 2. Although data from GPRC5D-related clinical trials need to be accumulated further, GPRC5D CAR-T cells have shown high ORR and CR rates, low incidence of ≥ grade 3 CRS and ICANS, and encouraging efficacy in patients who do not respond to or relapse after BCMA-targeted therapy. These results demonstrate that GPRC5D is a very potential immunotherapeutic target for R/R MM after BCMA.

## Data Availability

The material supporting the conclusion of this study has been included within the article.

## Abbreviations

BCMA: B cell maturation antigen
R/R: Relapsed or refractory
MM: Multiple myeloma
GPRC5D: G protein-coupled receptor, class C group 5 member D
CAR: Chimeric antigen receptor
ASH: American Society of Hematology
ORR: Overall response rate
CRS: Cytokine release syndrome
ICANS: Immune effector cell-associated neurotoxicity syndrome
BiTE: Bispecific T cell engager
AEs: Adverse events
IV: Intravenous
SC: Subcutaneous
iPSCs: Induced pluripotent stem cells
